# Could SCGF-Beta Levels Be Associated with Inflammation Markers and Insulin Resistance in Male Patients Suffering from Obesity-Related NAFLD?

**DOI:** 10.3390/diagnostics10060395

**Published:** 2020-06-11

**Authors:** Giovanni Tarantino, Vincenzo Citro, Clara Balsano, Domenico Capone

**Affiliations:** 1 Department of Clinical Medicine and Surgery, Federico II University Medical School of Naples, 80131 Naples, Italy; 2 Department of General Medicine, “Umberto I” Hospital, Nocera Inferiore (Sa), 84014 Nocera Inferiore, Italy; v.citro@libero.it; 3 Department of Clinical Medicine, Life, Health & Environmental Sciences-MESVA, University of L’Aquila, 67100 L’Aquila, Italy; clara.balsano@cc.univaq.it; 4 Clinical Pharmacology Consultant, Casoria, 80026 Naples, Italy; domenico.capo2016@gmail.com

**Keywords:** SCGF-β levels, insulin resistance, hepatic steatosis, obesity, CSFs

## Abstract

One of the pathologic hallmarks of obesity is macrophage infiltration of adipose tissue that has been confirmed as source of multipotent adult stem cells. Stem cell growth factor-beta (SCGF-β) shows activity on granulocyte/macrophage progenitor cells in combination with granulocyte macrophage colony-stimulating factor (GM-CSF) and macrophage colony-stimulating factor (M-CSF). Obesity-associated inflammation induces insulin resistance (IR), which is central to nonalcoholic fatty liver disease (NAFLD) or hepatic steatosis (HS). We searched for relationship between levels of SCGF-β and those of C-reactive protein (CRP), interleukin-6 (IL-6), tumor necrosis factor-β (TNF-β), interleukin-12p40 (IL-12p40), interleukin-10 (IL-10), ferritin, GM-CSF and M-CSF and between SCGF-β concentrations and IR in obese patients with HS. Eighty obese patients were retrospectively studied. Serum cytokines levels were appreciated by magnetic bead-based multiplex immunoassays. IR was evaluated by homeostatic model assessment (HOMA), HOMA-derived β-cell function (HOMA-B%), quantitative insulin sensitivity check Index (QUICKI) and single point insulin sensitivity estimator (SPISE). HS and spleen volume were assessed by ultrasonography (US). SCGF-β and IL-6 levels predicted HOMA values (*p* = 0.032 and 0.041, respectively) only in males. In male patients, CRP and IL-6 levels (*p* = 0.007) predicted SCGF-β concentrations (*p* = 0.03 and 0.007, respectively), which in turn predicted HS at US, *p* = 0.037. SCGF-β levels were linked to IR and HS severity with the mediation role of CRP. IL-10 levels negatively predicted SCGF-β concentrations (*p* = 0.033). M-CSF levels predicted serum concentration of both TNF-β and IL-12p40 (*p* = 0.00), but did not predict serum IL-10 (*p* = 0.30). Prediction of HOMA values by SCGF-β levels, likely mediated by markers of inflammation, characterizes this study, shedding some light on mechanisms inducing/worsening IR of male patients with obesity-related NAFLD.

## 1. Introduction

Macrophages are key mediators of obesity-induced insulin resistance (IR). This phenomenon is due to direct and paracrine signals from M1 classically-activated-macrophages while M2 alternatively-activated-macrophages fail to generate these effects [1,2,3]. Several studies emphasize the role of C-reactive protein (CRP) in promoting differentiation toward a pro-inflammatory M1 phenotype [4]. In this sense, chronic low-grade inflammation is revealed by high levels of serum interleukin-6 (IL-6). Furthermore, concentrations of TNF-β playing a critical role in cell-cell interactions and lymphocyte trafficking [5], of interleukin-12 subunit p40 (IL-12p40) acting as a chemoattractant for macrophages [6] and of interleukin-10 (IL-10), an anti-inflammatory cytokine [7], assess the inflammation severity. Finally, serum ferritin, a marker of cellular damage [8], and spleen volume [9] are reckoned as reliable indices of chronic inflammation.

Obesity-associated inflammation increases the risk of type 2 diabetes mellitus (T2DM), polycystic ovary syndrome (PCOS) and obstructive sleep apnea syndrome (OSAS) through induction of IR [10]. What is more, IR is an almost universal finding in obesity-related nonalcoholic fatty liver disease (NAFLD), further expression of metabolic syndrome (MS), leading to hepatocarcinoma [11].

Stem cell growth factor-beta (SCGF-β) shows activity on granulocyte/macrophage progenitor cells [12,13] in combination with other cytokines, i.e., granulocyte macrophage colony-stimulating factor (GM-CSF) and macrophage colony-stimulating factor (M-CSF), the former exhibits amplified response to M1 polarizing stimuli while the latter potentiates responses to M2 stimuli [14]. Recently, adipose tissue (AT) has been confirmed as a source of multi-potent adult stem cells [15].

However, little is known about the possible role played by SCGF-β in the metabolic derangement of obesity and its complications. With this in mind, we aimed at finding associations between SCGF-β levels and some molecules promoting or suppressing inflammatory responses in obese patients suffering from NAFLD or hepatic steatosis (HS). Specifically, we took into consideration serum levels of CRP, IL-6, TNF-β, IL-12p40, ferritin, IL-10, GM-CSF and M-CSF. But, the core investigation was to tackle any relationship of SCGF-β to IR as well as to the severity of HS.

## 2. Methods

### 2.1. Study Design

In this retrospective study we used the same patient sample contained in a previous research [16], according to the International Committee of Medical Journal Editors (ICMJE) at http://www.icmje.org/recommendations/browse/publishing-and-editorial-issues/overlapping-publications.html. 

Ethical standards: This study is exempted from ethical approval by ethics committee being a retrospective study conducted on already available data (for which formal consent may not be needed or is difficult to obtain).

### 2.2. Population

Data present in records of eighty obese patients, whose characteristics are reported below, were statistically analyzed.

### 2.3. Inclusion Criteria

Various grade-obesity with HS was the main phenotype of the examined population, characterized by low rate of co-morbidities such as T2DM and hypertension. Those subjects were characterized by a prevalently sedentary lifestyle.

### 2.4. Exclusion Criteria

Patients were excluded if, at the time of blood specimen collection, they self-reported present or antecedent (past month) influenza or cold status, or there had been history of unexplained weight loss in the past months (i.e., ±10% initial body weight) or recent illness/chronic disease, and the use of supplements or medications that might have affected metabolism or body homeostasis (e.g., steroids or NSAIDs).

Obese patients were not suffering from cardiac ischemia, cerebrovascular and peripheral artery diseases.

Co-morbidities were ruled out: OSAS in presence of loud habitual snoring, witnessed apnea, and excessive daytime sleepiness; PCOS on the basis of irregular or no menstrual periods, facial hair, acne, pelvic pain, difficulty getting pregnant; psoriasis on the appearance of skin, typically red, dry, itchy, and scaly; chronic kidney disease on the basis of blood and urine tests indicating renal failure.

Furthermore, any viral, autoimmune, metabolic liver disease was ruled out by using appropriate testing, according to well-accepted diagnostic guidelines. Celiac disease was excluded by evaluating IgA anti-tissue transglutaminase antibodies. Alcohol abuse was disallowed, following the DSM-IV diagnostic criteria, by means of screening tests such as MAST and CAGE, as well as random tests for blood alcohol concentration and the use of a surrogate marker, e.g., mean corpuscular volume.

### 2.5. Ultrasound Determinations

The spleen longitudinal diameter (SLD), assessed on longitudinal scan, (Esaote Genoa, Italy), was chosen to evaluate the spleen volume. The ultrasound (US) classification of HS was based on the following scale of increased echogenicity: grade 0 = absent, 1 = light, 2 = moderate, 3 = severe, unravelling the difference between the densities of the liver and the right kidney obtained on the same longitudinal sonographic plane. Visceral adipose tissue (VAT) was defined as the distance be-tween the anterior wall of the aorta and the internal face of the recto-abdominal muscle perpendicular to the aorta, measured one cm above the umbilicus. When the aortic walls were obscured by bowel gas, a Doppler scan was used to detect them [9].

### 2.6. Anthropometric Measures and Clinical Data

The class of obesity (I, II, III) was established according to the following BMI cut-offs, i.e., 30–34.9, 35–39.9 and >40 kg/m^2^, respectively. Visceral obesity was assessed measuring waist circumference (WC) at the midpoint between the lower border of the rib cage and the iliac crest. Hip circumference was measured around the widest part of the buttocks, with the tape parallel to the floor. Subsequently, the waist to hip ratio (WHR) was calculated. Systolic blood pressure and diastolic blood pressure were detected in a sitting position after resting for at least 5 min. Blood pressure were measured at least twice and then the average was calculated for analyses. T2DM was diagnosed if fasting venous blood glucose was ≥126 mg/mL or glycated hemoglobin was ≥6.5 in two determinations or 2 h venous blood glucose was 200 mg/mL or received glucose-lowering therapy.

### 2.7. Metabolic Assessment

The Adults Treatment Panel III was carried out to define the presence of MS, considering at least three criteria. Furthermore, the criteria for Europids of the International Diabetes Classification (IDF) were followed. IR was studied by a homeostatic metabolic assessment (HOMA), [17]. Two cut-offs of HOMA were introduced to assess the presence of IR, i.e., 2 [9], and 2.76 [18]. HOMA-derived β-cell function (HOMA-B%) was also measured, with range between 0.45 in healthy individuals and 0.30 in diabetics [19]. As surrogate of insulin sensitivity, the single point insulin sensitivity estimator (SPISE) was performed. A SPISE cutoff value lower than 6.61 indicated IR [20].

### 2.8. Laboratory Testing

Triglyceride values of subjects who had fasted at least 12/14 h were obtained, averaging the results of two determinations made on different days.

The normal range hs-CRP was between 0.03 and 0.86 mg/dL in men and between 0.02 and 0.91 mg/dL in women (BioCheck, Inc., South San Francisco, CA, USA). Contextually, ferritin levels were determined by an immunoturbidimetric assay. Briefly, human ferritin agglutinates with latex particles coated with anti-ferritin antibodies. The precipitate is determined turbidimetrically at 570/800 nm using Roche/Hitachi Cobas c systems [21], with well-accepted normal range for adult males and female of 20–250 and 10–120 ng/mL, respectively.

Human cytokines were measured by using Bio-Plex Pro™ Cytokine Assays (Biorad Laboratories, Inc., Hercules, CA, USA) The cytokines levels of 78 patients derived from a previously studied 48-cytokine/chemokine panel [22]. Thirty three healthy subjects had been previously evaluated to set the normal values of SCGF-β levels.

### 2.9. Statistics

Basal data, normally and not normally distributed, were presented as mean plus standard deviation (SD) or as median plus 25–75 interquartile range (IQR), respectively. The difference in medians was assessed by the two-sample Wilcoxon rank-sum (Mann–Whitney) test. The two-way cross-tabulation was studied by the Pearson correlation co-efficient (chi square). The Kruskal–Wallis equality-of-populations rank test was used when dealing with more than two variables.

Dealing with univariate analysis, the linear regression analysis was performed. When detecting the presence of outliers and in suspicion of heteroscedasticity, the correlation was analyzed by the robust regression. In one occasion, i.e., evaluating SLD as predictor, a regression that combines multiple replications with data simulation was applied (bootstrap method). An ordered probit model was employed to estimate predictions between an ordinal dependent variable, i.e., HS at US and a set of independent variables.

To highlight unobserved confounding variables two methods were adopted alternatively or combined according to the first output. The first one consisted in testing for mediation and was performed as a four step approach in which several regression analyses were performed; the significance of the coefficients were examined at each step to study the so-called indirect effect [23]. Complete mediation is present when the independent variable no longer influences the dependent variable after the mediator has been controlled and all of the above conditions are met. The second method took into account the instrumental variables (IV) to estimate causal relationships. The type of model was random effects and the estimator was Baltagi-Changone. At multiple linear regression the standardized beta (β) coefficient was calculated to compare the strength of each independent variable.

Addressing binary dependent variables the prediction tool carried out was the logistic regression by which the odds ratio with related 95% CI was evaluated.

In order to evaluate the degree of pair observations at US, the concordance correlation coefficient (ρc) was adopted to measure precision and accuracy.

The power of this study was calculated on the difference of means of SCGF-β levels between obese patients and control group. To further deepen this aspect, a power analysis was performed using a slope test in the linear regression between the SCGF-β levels and the HOMA values in the whole population of obese patients.

Stata 16.1, Copyright 1985–2019, StataCorp LLC, 4905 Lakeway Drive, College Station, Texas 77845-4512, USA, was the program on which we run statistics.

## 3. Results

### 3.1. Prevalence

The clinical, laboratory and instrumental characteristics of obese patients are shown in Table 1.

MS was present in 64% of this population. The obesity classes (I/II/ and III) did not show a different distribution according to gender, Pearson’s chi square, *p* = 0.74. WCs and WHRs were greater in females than males, *p* = 0.001 and 0.0007, respectively, two-sample Wilcoxon rank-sum (Mann–Whitney) test. Obese patients showed normal or slight elevated liver enzymes.

**Figure 1:**
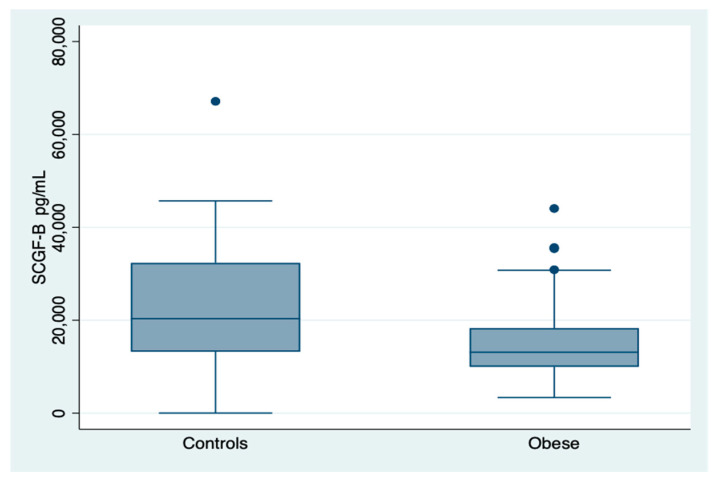
Distribution of stem cell growth factor-beta (SCGF-β) serum levels in the reference and the obese patients group. It is evident a reduction of SCGF-β levels in the obese patients group compared to reference values (controls) with a modest overlapping. A linear regression analysis was performed.

SCGF-β serum levels were 13.113 (9.976–18.299) pg/mL in this obese patients cohort, while were 29.247 (13,215.38–32,345.12) pg/mL) in subjects used as reference, with two-sample Wilcoxon rank-sum (Mann–Whitney) test evidencing significant lower median values of SCGF-β in the obese patients cohort (*z* = 3.471, *p* = 0.005), (Figure 1).

The levels of SCGF-β showed no difference throughout the various classes of obesity, chi-squared = 3.205, *p* = 0.2014, Kruskal–Wallis equality-of-populations rank test. The severity of HS (expressed as grades) also controlled for gender overlapped, Pearson chi square = 3.86, *p* = 0.145; anyway, HS was characterized by light or moderate grade.

The median level of M-CSF in obese patients was low, i.e., 17.3 (13.9–22) pg/mL. According to the Biorad Bullettin tech note 6029 available by entering on Google and searching the specific document, normal values of M-CSF ranged from 6.00 to 208.00, median 29.64 pg/mL. In addition, the concentrations of GM-CSF in obese patients were low, median 2 (0.14–18.7) pg/mL. In the same Bullettin, GM-CSF normal levels ranged between 3 and 122.00, median 6.78 pg/mL. There was a clear difference between these findings and the reference values of both CSFs.

Concerning the main inflammatory responses of pro-inflammation type, the median CRP concentration was 0.56 (0.27–1.30) mg/L, specifically in obese female patients 0.55 (0.34–1.38) and in obese male patients 0.59 (0.23–1.22), being only the values of the third quartile superior to the normal range in both genders.

The median level of IL-10 in obese patients was high, i.e., 11.65 (2.41–33) pg/mL with respect to the reference values, which ranged from 0.40 to 2, median 0.00 pg/mL, according to the aforementioned bulletin.

The median values of HOMA in every class of obese patients was overlapping, Kruskal–Wallis test, *p* = 0.38. There was no gender difference in HOMA determinations, *p* = 0.66, two-sample Wilcoxon rank-sum test, although the median value of females was greater.

The detailed distribution of HOMA values (cut-off > 2) showed that only the median value of the second and third quartile in both genders was above this stringent cut-off. Nevertheless, the classification by gender of the insulin resistant obese patients, when setting a cut-off of HOMA >2, identified 26 females and 29 males, while using a cut-off > 2.76 the categorization ended up in 19 females and 23 males. Thus, according to different cut-offs the prevalence varied significantly, Pearson’s chi square 24.9, *p* = 0.000 and 15.5, *p* = 0.000 for males and females, respectively.

The HS severity in males and females, evaluated as median grade, was not significantly different, *p* = 0.08, two-sample Wilcoxon rank-sum test.

### 3.2. Predictions of SCGF-β Levels by Indices of Inflammation in Obese Patients

**Figure 2:**
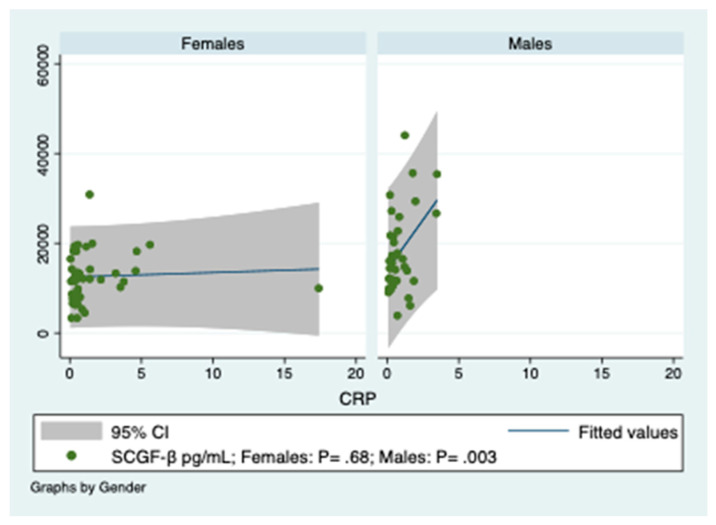
Prediction of SCGF-β serum concentrations by C-reactive protein (CRP) levels. CRP, C reactive protein. *Y*-axis reports SCGF-β serum concentrations. The regression line, obtained by linear regression, in the graph of males is sloped at an angle confirming that there is a strong relationship. CRP is expressed in mg/mL.

CRP concentrations significantly predicted SCGF-β levels only in obese male patients, (Figure 2).

Similarly, conveying a positive prediction, IL-6 serum levels were associated with SCGF-β serum concentrations in obese male patients. The serum determination of ferritin was predictor of SCGF-β levels in obese female patients, (Supplementary Figure S1).

To reinforce previous findings, SCGF-β serum gradient was negatively predicted by IL-10 concentrations. SCGF-β levels were not predicted by TNF-β levels, while were significantly associated to IL-12p40 levels in the whole population of obese patients. Intriguingly, SLD measurements predicted SCGF-β concentrations, (Supplementary Figure S2). The graph of this Figure (the large dispersion of values outside the 95% CI) prompted us to carry out a linear regression with bootstrap replications (200), obtaining a significant prediction, i.e., *p* = 0.047. Detailed regression statistics concerning the link between SCGF-β and markers of the inflammation milieu of the obese are summarized in Supplementary Table S1.

### 3.3. Predictions of SCGF-β Levels by Colony-Stimulating Factors

Dealing with the interaction between SCGF-β and both GM-CSF and M-CSF levels, it should be stressed that only M-CSF was predicted by SCGF-β, as evident in Supplementary Table S2.

A successive step consisted in tackling any prediction of the main cytokines, i.e., IL-6, TNF-β, IL-12p40 and IL-10 by M-SCF levels, being M-CSF, between CSFs, the only one found to be linked to SCGF-β. M-CSF concentrations predicted only TNF-β and IL-12p40, but not the pro/anti inflammation ones, in both males and females, with strong R-squared values, (Supplementary Table S3).

### 3.4. Prediction of HOMA by SCGF-β, M-CSF, TNF-β, IL-12p40, IL-6 and IL-10

Studying the prediction of IR presence, evaluated as HOMA values, by SCGF-β, M-CSF, TNF-β, IL-12p40, IL-6 and IL-10, except a weakly significant prediction by IL-6, the main forecast was due to SCGF-β levels only in males, as evident in Supplementary Table S4.

### 3.5. Prediction of SCGF-β Levels by the Four Surrogate Markers of Insulin Resistance

The different strength of prediction of SCGF-β levels by the four surrogate markers of IR studied in our population of obese patients is shown in Supplementary Table S5, evidencing the superiority of HOMA determination upon the remainders. Considering that SPISE is index of insulin sensitivity, a noticeable finding in the population of obese patients was the negative prediction of HOMA by SPISE, i.e., coefficient, standard error, t, P>|t|, 95% confidence interval of: −1.206, 0.5101, −2.36, 0.021, and −2.221 −0.190, respectively.

### 3.6. Prediction of Hepatic Steatosis by SCGF-β Levels and HOMA

**Figure 3:**
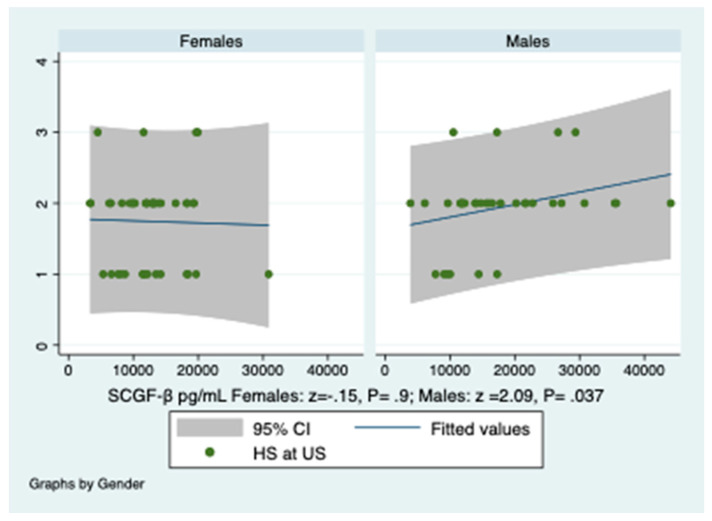
Prediction of the severity of hepatic steatosis at ultrasonography by SCGF-β levels. *Y*-axis reports the severity of hepatic steatosis. There is a gender-related difference of SCGF-β levels in predicting the severity of hepatic steatosis at ultrasonography, HS at US, expressed in grades (1–3). Ordered probit regression. A linear regression analysis was performed.

Turning the point versus HS, SCGF-β levels predicted its severity expressed in grades, only in males, as shown in Supplementary Table S6 and Figure 3.

Being IR, as repeatedly emphasized, central to NAFLD or HS, a successive step was to confirm the strict link between HOMA values and the liver disease severity, expressed as grades, in the obese, (Supplementary Table S7).

### 3.7. Tackling the Problem of Unobserved Confounding Variables

To test the presence of CRP as possible covariate, when SCGF-β levels predicted HOMA values in obese patients, the results of two methods are evidenced in Supplementary Tables S8 and S9, confirming the full mediation of CRP. Nevertheless, evaluating whether HOMA values were a full or partial mediator between SCGF-β and HS, we found that they should be considered as partial mediator, casting some doubts on the direction of its effect, as it is observable in Supplementary Table S10.

As an interesting report, the IR presence predicted the MS occurrence, with the following output at the logistic regression: odds ratio, standard error, z, P>|z| and 95% confidence interval of: 8.702, 4.831, 3.90, 0.000, and 2.931–25.837, respectively. The IR presence with a HOMA cut-off > 2 was predicted by WC, i.e., |odds Ratio 1.05, P>|z|= 0.024 [95% confidence interval = 1.006 1.093]. Again, the IR presence, tested by a HOMA cut-off > 2.76 (specifically, that found in a population of healthy Italian subjects) was predicted by VAT, i.e., |odds ratio: 1.29, *p* = 0.015, 95% confidence interval = 1.05–2.6.

The intra/inter-observational variability of US estimations was not significant, the mean difference being 1.9 and 3.1%, and 2.3 and 3.1% for the HS and VAT, respectively, with an ρ_c_ of 0.91.

The power of this research was carried out by measuring the difference of means and SDs of SCGF-β in obese patients and the reference group, i.e., 14,964.51 ± 7,796.883 and 23,726.5 ± 13,976.39. The study turned out to be sufficiently powered (alpha = 0.05, power = 0.85) considering the sample size of the two retrospectively evaluated cohorts (estimates sample size: total *n* = 64, *n* = 32 for group). Furthermore, the power analysis for a slope test in a simple linear regression, with a significant level of 0.05 and a power of 0.80 gave as estimated sample size *n* = 34.

## 4. Discussion

IR may depend on excessive fat volume, while inflammation may depend on local secretion of inflammatory molecules [22,24]. In our study CRP levels, adjusted for gender according to previous research [25], ended up in being increased only in one third of obese patients, showing an association with SCGF. IL-6 levels predicted SCGF-β concentrations only in males. In the same patients SCGF-β levels predicted HOMA values.

The pro-inflammatory cytokines including M-CSF, TNF-β, IL-12p40, IL-6 were not associated with HOMA values, except a prediction by IL-6, showing that the chronic inflammation status was of low-grade. This hypothesis is reinforced by the modest increase of CRP levels.

In keeping with evidence that CRP impacts on the anti-inflammatory/pro-inflammatory balance accentuating inflammation [26], we speculate that scarcely elevated CRP levels could make IL-10 more available, in an attempt to partially reduce inflammation, the main driver of IR. In this respect, we would like to draw the attention on our findings, i.e., IR is present in near half of obese patients, high levels of IL-10 and the protective response of IL-12p40 [6]. Alternatively serum concentrations of SCGF-β could resulting from a scarce autocrine/paracrine function of hematopoietic stem/progenitor cells [27]. It is believed that, by switching M1 to M2, inflammation can be reversed and IR can be ameliorated [28].

There has been reported gender-difference of HS severity [29,30]. We found that patients with a more pronounced HOMA had a higher prevalence of moderate and severe steatosis compared to those with HOMA below the median, as reported [31], even though our median HOMA values overlapped according to gender. The fact that SCGF-β levels predicted only in males the severity of HS, could show that the adiposity of these subjects impact on the inflammation status and/or on immune system. Accordingly, CRP levels and those of IL-6 predicted SCGF-β concentrations only in males. These results agree with the finding that only in males SCGF-β levels predict IR, evaluated as HOMA. The mediating role of CRP is plausible if we take into account its functional role in inflammation [32], in addition being a marker of this process. About the surprising finding that ferritin concentrations predicted SCGF-β levels in females alone, it should be pointed out that ferritin is an index of inflammation but also of iron status and that these conditions often overlap. The limitations of prediction (its bi-directionality) should be stressed. Furthermore, ethical and technique difficulties warned us to perform liver biopsy.

In conclusion, the prediction of HOMA values by SCGF-β levels, likely mediated by inflammation, characterizes this study, shedding light on mechanisms inducing/worsening IR of male patients with obesity-related NAFLD.

## Figures and Tables

**Table 1:** Patient baseline data (clinical, laboratory and instrumental).

Age (years)	46 (34–53) *	Gender M/F (*n*)	36/44
Obesity classes I/II/III (*n*)	8/26/46	MS (APT III) Yes/Not (*n*)	51/29
		MS (IDF) Yes/Not (*n*)	51/29
T2DM (*n*)	16	OSAS/PCOS (*n*)	0/0
Hypertension (*n*)	4	ACV/CCV disease (*n*)	0/0
HOMA-B%	37.11 (22.4–49.8) *	HOMA Total	2.78 (1.85–4.18) *
HOMA (M)	2.75 (1.9–4.7) *	HOMA (F)	3–12 (1.55–4.18) *
SPISE	6.6 ±1.7 °	QUICKI	0.32 (0.31–0.35) *
GM-CSF (pg/mL)	2 (0.14–18.7) *	M-CSF	17.3 (13.9–22) *
SCGF-β (pg/mL)	13,113 (9976–18,299) *	TNF-β (pg/mL)	2.7 (0.21–6.2) *
CRP (M) (mg/L)	0.59 (0.23–1.22) *	IL-12p40 (pg/mL)	234 (130–317) *
CRP (F) (mg/L)	0.55 (0.34–1.38) *	IL-6 (M) (pg/mL)	4.3 (1.6–15.2) *
Ferritin (M) (ng/mL)	167.5 (85–234.5) *	Il-6 (F)(pg/mL)	6.4 (3–19.3) *
Ferritin (F) (ng/mL)	41.5 (20–69) *	IL-10 (pg/mL)	11.6 (2.4–33.1) *
IR > 2 (M) (*n*)	26	IR > 2 (F) (*n*)	29
IR > 2.76 (M) (*n*)	19	IR > 2.76 (F) (*n*)	23
HS at US grades 1/2/3	22/50/8	SLD cm	11.0 (10.2–12.4) *
ALT (U/I)	28 (21.5–39) *	Gamma-GT U/I	25 (16.5–42.5) *

Data expressed as median (IQR) are labelled by the symbol * while data expressed as mean ± SD by the symbol °. M, males; F, females; yes/not, present/absent; *n*, number; MS, metabolic syndrome; T2DM, Type 2 diabetes mellitus; OSAS, obstructive sleep apnea syndrome; PCOS, polycystic ovary syndrome; ACV/CCV, acute/chronic cardiovascular disease; IR, insulin resistance, HS at US, hepatic steatosis at ultra-sonography, SLD, spleen longitudinal diameter; IDF, International Diabetes Federation; HOMA, homeostatic model assessment; SPISE, single point insulin sensitivity estimator; QUICKI, quantitative insulin sensitivity check Index; GM-CSF, granulocyte macrophage colony-stimulating factor; M-CSF, macrophage colony-stimulating factor; SCGF, Stem cell growth factor; TNF, tumor necrosis factor; CRP, C-reactive protein; IL, interleukin; ALT, alanine aminotransferase; GT, glutamyl transferase.

## Supplementary Materials

The following are available online at https://www.mdpi.com/2075-4418/10/6/395/s1.
