# Supplementary material for: Could SCGF-Beta Levels Be Associated with Inflammation Markers and Insulin Resistance in Male Patients Suffering from Obesity-Related NAFLD?
Source: Diagnostics (Basel). 2020 Jun 11;10(6):395. doi: 10.3390/diagnostics10060395 (PMC7345627; doi:10.3390/diagnostics10060395)
Supplement: Supplementary file 1 [file diagnostics-10-00395-s001.pdf]

Supplementary Materials

Could SCGF-Beta Levels Be Associated with Inflammation Markers and Insulin Resistance in Male Patients Suffering from Obesity-Related NAFLD?

Giovanni Tarantino, Vincenzo Citro, Clara Balsano and Domenico Capone

1. Introduction

Indeed, there is a reduced group of studies appearing in literature concerning different settings and what is more, they are characterised by a surprising variability of the serum concentrations of this growth factor. For example, serum levels of SCGF-β ranged in patients undergoing bone marrow transplantation from 9760 ± 6810 to 25,010 ± 15,140 pg/mL [1]. Still, different levels of this cytokine were found in unstable asymptomatic carotid plaques compared to stable plaques, varying from undetectability to levels of 600 pg/mL [2]. Furthermore, SCGF-β was significantly increased in patients suffering from Chagas' disease with advanced heart failure compared to those without heart failure, exceeding 22,940 ± 2638 pg/mL, [3]. Recently, authors demonstrate that levels > 21,000 pg/mL) of serum SCGF-β are associated with non responsiveness to therapy of HCC [4]. SCGF-β is elevated in the circulation of patients with chronic spinal cord injury confronted with uninjured subjects, i.e., 47,037 pg/mL vs. 35,521 pg/mL [5]. Finally, Schirmer et al. found in plasma samples from human collateral circulation a median (interquartile) value of SCGF-β equal to 2624.00 (1646.38) pg/mL [6].

2. Aim

Considering that AT participates in inflammatory pathways [7] and recruitment of macrophages into AT involves interactions of innate and adaptive immunity in multiple organs, although the crosstalk between adipocytes and macrophages lays at its core [8,9], we asked ourselves whether SCGF-β could have a direct or indirect role in a new AT environment characterised by an inflammatory status, leading to IR.

3. Results

Table S1. Predictions of SCGF-β levels by indices of inflammatory responses. It is noteworthy that CRP is the stronger predictor, while IL-10 negatively predicted SCGF-β; d.v., dependent variable; i.v., independent variable. In bold are highlighted the significant ones. The low R-squared in presence of significance shows that even noisy, high-variability data can have a significant trend. The trend indicates that the predictor variable still provides information about the response even though data points fall further from the regression line in graph.

**Linear regression, Robust**					
**Females, Number of obs=43**	**R-squared=0.028**				
d.v. SCGF-β	Coef.	Std. Err.	t	P>|t|	[95% Conf. Interval]
i.v. CRP .	102.755	205.0394	0.42	0.677	-392.112/597.6221
**Males, Number of obs=35**	**R-squared=0.17**				
d.v. SCGF-β	Coef.	Std. Err.	t	P>|t|	[95% Conf. Interval]
i.v. CRP .	4453.402	1413.839	**3.15**	0.003	-1576.925/7329.878

Linear regression, Robust, Bootstrap replications=200 Number of obs=78

R-squared=0.0480

d.v. SCGF-β	Coef.	Std. Err.	z	P>|z|	[95% Conf. Interval]
i.v. SLD .	1119.491	564.2516	1.98	0.047	-13.57866/2225.40

Linear regression, Robust

Females, Number of obs=43 **R-squared=0.1422**

d.v. SCGF-β	Coef.	Std. Err.	t	P>|t|	[95% Conf. Interval]
i.v. Ferritin .	49.10791	23.07629	**2.13**	0.039	-2.504392/95.71143

Males, Number of obs=35 **R-squared=0.039**

d.v. SCGF-β	Coef.	Std. Err.	t	P>|t|	[95% Conf. Interval]
i.v. Ferritin .	11.3087	11.01974	1.03	0.312	-11.11114/33.72853

Linear regression, Robust **Number of obs=78** **R-squared=0.021**

d.v. SCGF-β	Coef.	Std. Err.	t	P>|t|	[95% Conf. Interval]
i.v. IL-10	18.28091	8.415729	**-2.17**	0.033	-.35.04229/1.519538

Linear regression, Robust

Females, Number of obs=43 **R-squared=0.0020**

d.v. SCGF-β	Coef.	Std. Err.	t	P>|t|	[95% Conf. Interval]
i.v. IL-6 .	14.78666	64.70944	0.23	0.820	-115.8967/145.47

Males, Number of obs=35 **R-squared=0.1835**

d.v. SCGF-β	Coef.	Std. Err.	t	P>|t|	[95% Conf. Interval]
i.v. IL-6 .	205.8147	71.15122	**2.89**	0.007	61.05648/350.573

Linear regression, OLS **Number of obs=78** **R-squared=0.0644**

d.v. SCGF-β	Coef.	Std. Err.	t	P>|t|	[95% Conf. Interval]
i.v. IL-12p40	14.06439	6.148093	2.29	0.025	1.819398/26.30938

Linear regression, Robust **Number of obs=78** **R-squared=0.0059**

d.v. SCGF-β	Coef.	Std. Err.	t	P>|t|	[95% Conf. Interval]
i.v. TNF-β	118.0296	150.0715.	0.79	0.434	-180.8637/416.9229

Table S2. Predictions of SCGF-β levels by colony-stimulating factors. SCGF-β predicts only M-CSF; d.v., dependent variable; i.v., independent variable. In bold are highlighted the significant ones. The low R-squared in presence of significance shows that even noisy, high-variability data can have a significant trend. The trend indicates that the predictor variable still provides information about the response even though data points fall further from the regression line in graph.

**Linear regression, OLS**	**Number of obs=78**		**R-squared=0.047**		
d.v. GM-CSF	Coef.	Std. Err.	t	P>|t|	[95% Conf. Interval]
i.v. SCGF-β	0.0001142	0.0003037	0.38	0.708	0.0004906/0.000719

**Linear regression, Robust**	**Number of obs=78**		**R-squared=0.083**		
d.v. M-CSF	Coef.	Std. Err.	t	P>|t|	[95% Conf. Interval]
i.v. SCGF-β	0.0002311	0.0000867.	**2.67**	0.009	0.0000584/0.0004038

Table S3. Prediction of M-CSF serum levels by Interleukin- 6, IL-12p40, TNF-β and IL-10. M-CSF levels predicted only cytokines involved in monocyte/macrophage recuritment and not the pro/anti inflammation ones; d.v., dependent variable; i.v., independent variable. In bold are highlighted the significant ones. The only low R-squared presented shows that even noisy, high-variability data can have a significant trend. The trend indicates that the predictor variable still provides information about the response even though data points fall further from the regression line in graph. On the contrary, the high R-squares signify that data are close to the fitted regression line, explain that this model explains more than more than a quarter of total variability.

**Linear regression, Robust,**	**Number of obs=78**		**R-squared=0.0033**		
d.v. IL-6	Coef.	Std. Err.	t	P>|t|	[95% Conf. Interval]
i.v. M-CSF .	14.21686	1.990788	0.49	0.624	-0.4977176/0.8242979

**Linear regression, Robust**	**Number of obs=78**		**R-squared=0.40**		
d.v. IL-12p40	Coef.	Std. Err.	t	P>|t|	[95% Conf. Interval]
i.v. M-CSFIL-10	14.21686	1.990788	**7.14**	0.000	10.25186/18.18186

**Linear regression, Robust**	**Number of obs=78**		**R-squared=0.335**		
d.v. TN-β	Coef.	Std. Err.	t	P>|t|	[95% Conf. Interval]
i.v. M-CSF	0.4711392	0.1089992	**4.32**	0.000	0.2540485/0.68823

**Linear regression, Robust**	**Number of obs=78**		**R-squared=0.046**		
d.v. IL-10	Coef.	Std. Err.	t	P>|t|	[95% Conf. Interval]
i.v. M-CSF	-0.6721816	0.6462692	-1.04	0.302	-1.959338/0.6149752

Table S4. Prediction of HOMA by SCGF-β, M-CSF, TNF-β, IL-12p40, IL-6 and IL-10. Apart the prediction of HOMA by IL-6, SCGF-β predicted sufficiently insulin resistance, evaluated as HOMA. On the basis of the prediction of HOMA by IL-6 the evaluation of a confounding variable, i.e., CRP was carried out, see Supplementary Table S9. The low R-squared shows that even noisy, high-variability data can have a significant trend. The trend indicates that the predictor variable still provides information about the response even though data points fall further from the regression line in graph; d.v., dependent variable; i.v., independent variable. In bold are highlighted the significant ones.

**Linear regression, Robust**					
**Females, Number of obs=43**	**R-squared=0.0243**				
d.v. HOMA	Coef.	Std. Err.	t	P>|t|	[95% Conf. Interval]
i.v. SCGF-β .	0.0000562	0.0000498	1.13	0.265	-0.0000443/0.0001567
**Males, Number of obs=35**	**R-squared=0.1537**				
d.v. HOMA	Coef.	Std. Err.	t	P>|t|	[95% Conf. Interval]
i.v. SCGF-β .	0.0002282	0.0001018	**2.24**	0.032	0.0000211/0.0004353

**Linear regression, Robust,**		**Number of obs=78**	**R-squared=0.64**		
d.v. HOMA	Coef.	Std. Err.	t	P>|t|	[95% Conf. Interval]
i.v. M-CSF .	-0.0505071	0.0942935	-0.54	0.594	-0.2383088/0.1372946

**Linear regression, Robust**		**Number of obs=78**	**R-squared=0.013**		
d.v. HOMA	Coef.	Std. Err.	t	P>|t|	[95% Conf. Interval]
i.v. TNF-β	-0.0284342	0.0623148	-0.46	0.649	-01525448/0.0956765

**Linear regression, Robust**		**Number of obs=78**	**R-squared=0.058**		
d.v. HOMA	Coef.	Std. Err.	t	P>|t|	[95% Conf. Interval]
i.v. IL-12p40	-0.0021346	0.0034262	-0.62	0.535	-0.0089584/0.0046892

**Linear regression, Robust**		**Number of obs=78**	**R-squared=0.084**		
d.v. HOMA	Coef.	Std. Err.	t	P>|t|	[95% Conf. Interval]
i.v. IL-6	0.0592377	0.0285458.	**2.08**	0.041	0.0024073/0.116068

**Linear regression, Robust**		**Number of obs=78**	**R-squared=0.020**		
d.v. HOMA	Coef.	Std. Err.	t	P>|t|	[95% Conf. Interval]
i.v. IL-10	0.0027228	0.0032282.	0.84	0.402	-0.003704/0.0091497

Table S5. Prediction of SCGF-β levels by the four surrogate markers of insulin resistance. It is clear that the best predictor of SCGF-β levels is HOMA, among other surrogate markers of insulin resistance; d.v., dependent variable; i.v., independent variable. In bold are evidenced the significant ones or the value (Beta) of greater effect.

**Multiple regression, Robust**		**Number of obs=78**	**R-squared=0.3063**		
d.v. SCGF-β	Coef.	Std. Err.	t	P>|t|	[95% Conf. Interval]
i.v. QUICKI .	110301.1	39213.64	**2.81**	0.006	-32148.41/188453.7
i.v. SPISE	-147.8333.	595.6343.	-0.25	0.805|	-1334.931/1039.264
i.v. HOMA-%B	.58.91907	18.69706	3.15	0.002	21.65589/96.18225
i.v. HOMA	1108.485	265.0487	**4.10**	0.000	580.2434/163726

Beta of QUICKI=0.5; Beta of SPISE=-0.03; Beta of HOMA-B%=0.37; **Beta of HOMA=0.56**

Table S6. Prediction of the hepatic steatosis severity by SCGF-β levels. There is gender-related difference in the prediction of hepatic steatosis severity at ultrasonography (HS at US) by SCGF-β levels; d.v., dependent variable; i.v., independent variable. In bold is evidenced the significant one.

Ordered probit regression, Robust

**Females, Number of obs=43**		**Pseudo R2=0.0004**			
d.v. HS at US	Coef.	Std. Err.	z	P>|z|	[95% Conf. Interval]
i.v. SCGF-β .	-5.62e-06	0.000038	0.15	0.882	-0.0000801/0.0000689
**Males, Number of obs=35**		**Psudo R2=0.0624**			
d.v. HS at US	Coef.	Std. Err.	z	P>|z|	[95% Conf. Interval]
i.v. SCGF-β .	0.0000436	0.0000209	**2.09**	0.037	2.65e-06/0.0000845

Table S7. Prediction of hepatic steatosis at ultrasonography by HOMA. HS at US, hepatic steatosis at ultrasonography; d.v., dependent variable; i.v., independent variable. In bold are evidenced the significant ones. Interestingly, HOMA predicted HS at US both in males and females; d.v., dependent variable; i.v., independent variable. In bold are highlighted the significant ones. By the way, the predicted values do not depend on the order of predictors in the equation, in the sense that we are always solving the same equation. It is useful to compare Table 8: CRP as eventual mediator between SCGF-β and HOMA.

Ordered probit regression, Robust

**Number of obs=80**	**Pseudo R2=0.0888**				
d.v. HS at US	Coef.	Std. Err.	z	P>|z|	[95% Conf. Interval]
i.v. HOMA .	0.1283419	0.0319009	**4.02**	0.000	0.0658173/0.1908665

**Females, Number of obs=44**		**Pseudo R2=0.2773**			
d.v. HS at US	Coef.	Std. Err.	z	P>|z|	[95% Conf. Interval]
i.v. HOMA .	0.5390745	0.1153244	**4.67**	0.000	0.3130427/0.7651062
**Males, Number of obs=36**		**Psudo R2=0.0544**			
d.v. HS at US	Coef.	Std. Err.	z	P>|z|	[95% Conf. Interval]
i.v. HOMA .	0.0696083	0.0334134	**2.08**	0.037	0.0041193/0.1350973

Table S8. Testing CRP as confounding variable between SCGF-β and HOMA. Mediation method. Because at multiple regression HOMA, controlled for CR, predicts no more SCGF-β, this statistical output tells us that CRP is a full mediator in this prediction; d.v., dependent variable; i.v., independent variable. In bold are evidenced the significant ones. It is interesting to note that the Betas of HOMA and CRP are quite similar.

Linear regression, Robust

Males. Number of obs=35

d.v. HOMA		Coef.	Std. Err.	t	P>|t|	[95% Conf. Interval]
i.v. SCGF-β	.	0.0002486	0.0001024	**2.43**	**0.021**	0.0000394/0.0004577

d.v. HOMA		Coef.	Std. Err.	t	P>|t|	[95% Conf. Interval]
i.v. CRP	.	3.15536 1.096902	**2.88**	**0.007**	0.9182133/5.392506	

d.v. SCGF-β		Coef.	Std. Err.	t	P>|t|	[95% Conf. Interval]
i.v. CRP	.	4747.25	1386.377	**3.42**	**0.002**	1915.891/7578.61

Multiple regression, Robust

Males, Number of obs=35

d.v. SCGF-β		Coef.	Std. Err.	t	P>|t|	[95% Conf. Interval]
i.v. HOMA		420.0378	401.1495	**1.05**	**0.303**	-397.077/1237.152
i.v. CRP	.	3097.937	2071.708	**1.50**	**0.145**	-1121.995/7317.868

Beta of HOMA=0.24; Beta of CRP=0.28

Table S9. Testing CRP as confounding variable between SCGF-β and HOMA. The method of Instrumental Variables (IV) to test confounding variables. A valid instrument (SCGF-β) induces changes (inversion of sign or no significance) in the explanatory variable (covariate, CRP, z = 0.41) but has no independent effect on the dependent variable (HOMA, z = 0.73), allowing to uncover the causal effect of the explanatory variable (CRP) on the dependent variable (HOMA). The strength of instrument is weighted by the following: F-statistic (Wald chi square) against the null (that the excluded instruments were irrelevant in the first-stage regression) it should be larger than Staiger and Stock's Rule of thumb (1997), i.e., less than ten; d.v., dependent variable; e.v., explanatory variable; ins.v., instrumental variable. This statistical output confirms the report of the multiple regression shown in Supplementary Table S10.

xtivreg HOMA SCGF-β (CRP= Age), be vce (robust).

Between-effects IV regression: Number = 78
Group variable: SCGF-β Number of groups = 77
R-sq: Obs per group: within= 1.0000; min= 1 between= avg= 1.0; overall= 0.0331 max = 0.0153
Wald chi²(2) =3.15; sd(u_i + avg (e_i.))= 9.304895 Prob > chi² = 0.2074
(Std. Err. adjusted for 77 clusters in SCGF-β)

d.v. HOMA	Coef.	Std. Err.	z	P>|z|	[95% Conf. Interval]
e.v. CRP	3.922337	9.584752	**0.41**	**0.682**	-14.86343/22.70811
ins.v. SCGF-β	.0.000134	0.0001839	**0.73**	**0.466**	-0.0002265/0.0004945

{p 0 16-17}Instrumented: CRP{p_end}
{p 0 16 -17}Instruments: SCGF-β Age {p-end}

Table S10. HOMA as partial mediator between SCGF-β and HS at US. HOMA values predicted the severity of hepatic steatosis at ultrasonography, HS at US, although to a lesser extend respect to the prediction of SCGF-β levels. A some form of mediation is supported remaining the effect of mediator (HOMA. herein in second output) significant after controlling for the independent variable, i.e., SCGF-β in multiple regression (herein in the third output). HOMA increased its significance in predicting HS at US with a difference in Coef. respect to that of the univariate analysis (herein the second output) of 43%. This last datum triggers an interesting debate, in the sense that we do not know for sure whether insulin resistance, evaluated as HOMA, was cause or effect of hepatic steatosis; d.v., dependent variable; i.v., independent variable. In bold are highlighted the significant ones or the value (Beta) of greater effect.

Linear regression, Robust

Males. Number of obs=35 **R-squared=0.1537**

d.v. HOMA		Coef.	Std. Err.	t	P>|t|	[95% Conf. Interval]
i.v. SCGF-β	.	0.0002282	0.0001018	**2.24**	0.032	0.0000211/0.0004353

d.v. HS at US	Coef.	Std. Err.	t	P>|t|	[95% Conf. Interval]
i.v. HOMA	.	0.0285048	0.0138959	**2.05**	0.048	0.0002649/0.0567447

Multiple regression, Robust

Males, Number of obs=35

d.v. HS at US	Coef.	Std. Err.	t	P>|t|	[95% Conf. Interval]
i.v. SCGF-β		4.37e-068.89e-060.49	0.624	-0.0000133/0.0000221	
i.v. HOMA	.	0.0521923	0.0145081	**3.60**	0.001	0.0232908/0.0810939

Beta of SCGF-β=0.5; Beta of HOMA=0.35

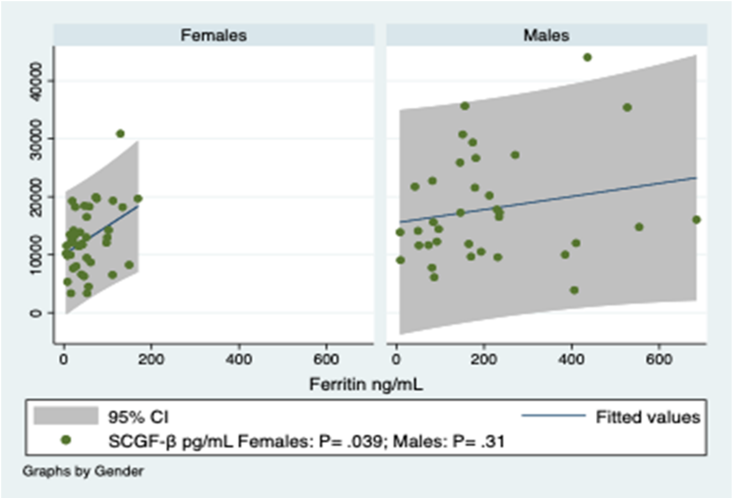

Figure S1. Prediction of SCGF-β serum concentrations by Ferritin levels. It is evident the significant prediction of ferritin concentrations versus SCGF-β levels only in females.

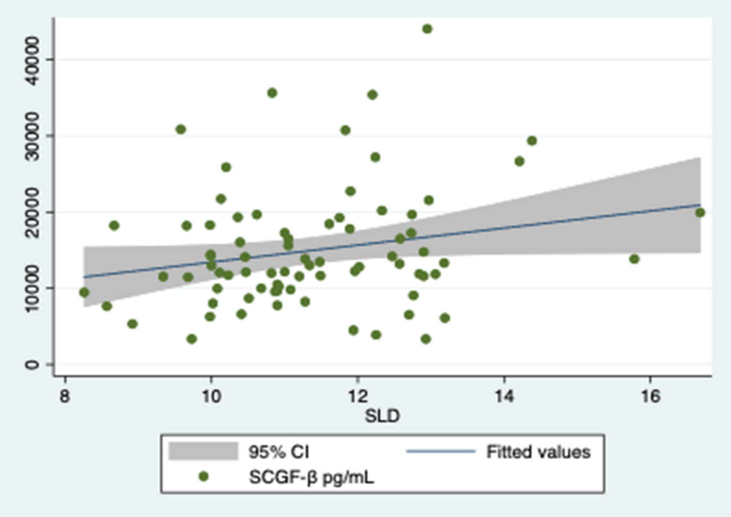

Figure S2. Prediction of SCGF-β levels by the spleen volume determinations. SLD, spleen longitudinal diameter at ultrasonography; It should be noted the large dispersion of values outside the 95% CI.

4. Conclusion

In other words, this is a possible example of an immunometabolic regulation. Anyway, it is still the case for expecting more confirmation from other studies, mainly on the side of gender difference, beyond a more compelling one from a purely mechanistic standpoint to give our hypotheses a greater construct.

5. Future directions

Being chronic inflammation a major factor in obesity and related co-morbidities, the hope is that some of the specific mechanisms could translate to optimising immune function in the obese during ageing in order to improve their health.

6. Methods

Measuring statistical associations, we chose a very powerful technique, i.e., regression, https://s3-eu-west-1.amazonaws.com/.../chapter_summary_ch13, which is used to identify the strength of the effect that independent variables have on a dependent variable. By the way, the predicted values do not depend on the order of predictors in the equation, in the sense that we are always solving the same equation. The statistical associations were performed separately on males and females, but presented as unique group or separate groups according to their significance.

References

1. Ito, C.; Sato, H.; Ando, K.; Watanabe, S.; Yoshiba, F.; Kishi, K.; Furuya, A.; Shitara, K.; Sugimoto, S.; Kohno, H.; et al. Serum stem cell growth factor for monitoring hematopoietic recovery following stem cell transplantation. *Bone Marrow Transplant.* **2003**, *32*, 391–398.
2. Schiro, A.; Wilkinson, F.L.; Weston, R.; Smyth, J.V.; Serracino-Inglott, F.; Alexander, M.Y. Elevated levels of endothelial-derived microparticles, and serum CXCL9 and SCGF-β are associated with unstable asymptomatic carotid plaques. *Sci. Rep.* **2015**, *5*, 16658.
3. Wang, Y.; Khan, A.; Heringer-Walther, S.; Schultheiss, H.P.; Moreira, M.; Walther, T. Prognostic value of circulating levels of stem cell growth factor beta (SCGF beta) in patients with Chagas' disease and idiopathic dilated cardiomyopathy. *Cytokine* **2013**, *61*, 728–731.
4. Sukowati, C.H.C.; Patti, R.; Pascut, D.; Ladju, R.B.; Tarchi, P.; Zanotta, N.; Comar, M.; Tiribelli, C.; Crocè, L.S. Serum Stem Cell Growth Factor Beta for the Prediction of Therapy Response in Hepatocellular Carcinoma. *Biomed. Res. Int.* **2018**, *2018*, 6435482.

5. Stein, A.; Panjwani, A.; Sison, C.; Rosen, L.; Chugh, R.; Metz, C.; Bank, M.; Bloom, O. Pilot study: Elevated circulating levels of the proinflammatory cytokine macrophage migration inhibitory factor in patients with chronic spinal cord injury. *Arch. Phys. Rehabil.* **2013**, *94*, 1498–1507.
6. Schirmer, S.H.; van Royen, N.; Moerland, P.D.; Fledderus, J.O.; Henriques, J.P.; van der Schaaf, R.J.; Vis, M.M.; Baan, J. Jr.; Koch, K.T.; Horrevoets, A.J.G.; et al. Local cytokine concentrations and oxygen pressure are related to maturation of the collateral circulation in humans. *J. Am. Coll. Cardiol.* **2009**, *53*, 2141–2147.
7. Weisberg, S.P.; McCann, D.; Desai, M.; Rosenbaum, M.; Leibel, R.L.; Ferrante, A.W. Obesity is associated with macrophage accumulation in adipose tissue. *J. Clin. Invest.* **2003**, *112*, 1796–1808.
8. Bai, Y.; Sun, Q. Macrophage recruitment in obese adipose tissue. *Obes. Rev.* **2015**, *16*, 127–136.
9. Geissmann, F.; Manz, M.G.; Jung, S.; Sieweke, M.H.; Merad, M.; Ley, K. Development of monocytes, macrophages, and dendritic cells. *Science* **2010**, *327*, 656–661.
